# A Scoping Review of Cognitive Screening Among Older Adults With Heart Disease

**DOI:** 10.1093/geroni/igaf122.3873

**Published:** 2025-12-31

**Authors:** Christopher Griffith, Lindsey Nelson, Helen Graham

**Affiliations:** University of Colorado Colorado Springs, Colorado Springs, Colorado, United States; University of Colorado Colorado Springs, Colorado Springs, Colorado, United States; UCCS, University of Colorado Colorado Springs, Colorado, United States

## Abstract

Strong associations exist between cognitive impairment (CI) and heart disease in older adults, and such conditions are often comorbid. Early identification of CI can improve health outcomes. However, standard screening protocols are lacking, and it is uncertain whether healthcare providers routinely conduct formal or informal assessments for CI. The purpose of this review was to better understand the extent to which healthcare providers screen for CI in older adults with heart disease within clinical settings and to identify which screening methods are used. A scoping review was conducted using Arksey and O’Malley’s systematic framework. Articles were included if they featured information about frequency, application, or other contextual factors about CI screening for adults with heart disease in clinical settings. Of 876 potential sources identified, 9 were included for the full review. The articles represented international screening practices, including the United States, Australia, the Netherlands, China, and Uganda. Most articles (n = 6) assessed healthcare providers’ practices and perspectives on cognitive screening. Results included a range of providers (e.g., nurses, psychologists, rehabilitation specialists, cardiologists) who routinely screen, 3% to 39%, and various screening measures (e.g., Montreal Cognitive Assessment, Mini-Mental Status Exam, Mini-Cog, and more). Barriers to regular screening were feasibility concerns and provider-specific attitudes and beliefs, among others. Recommendations included implementation of standardized screening protocols, increasing providers’ awareness of age-related considerations, and greater interdisciplinary collaboration, among others. Findings demonstrated that some providers recognize the importance of routine cognitive screening, but many lack adequate knowledge, training, and logistical support.

